# Evidence-Guided Diagnostic Reasoning for Pediatric Chest Radiology Based on Multimodal Large Language Models

**DOI:** 10.3390/jimaging12030111

**Published:** 2026-03-06

**Authors:** Yuze Zhao, Qing Wang, Yingwen Wang, Ruiwei Zhao, Rui Feng, Xiaobo Zhang

**Affiliations:** 1 College of Biomedical Engineering, Fudan University, Shanghai 200433, China; yzzhao23@m.fudan.edu.cn; 2 National Children’s Medical Center, Children’s Hospital of Fudan University, Shanghai 201102, China; 22211240021@m.fudan.edu.cn (Q.W.); yingwenwang@fudan.edu.cn (Y.W.); fengrui@fudan.edu.cn (R.F.); 3 Fudan Zhangjiang Institute, Shanghai 200120, China; 4 Shanghai Key Laboratory of Intelligent Information Processing, College of Computer Science and Artificial Intelligence, Fudan University, Shanghai 200433, China

**Keywords:** medical image diagnosis, chest X-ray, multi-modal diagnosis, multimodal large language model, pediatric disease diagnosis

## Abstract

Pediatric respiratory diseases are a leading cause of hospital admissions and childhood mortality worldwide, highlighting the critical need for accurate and timely diagnosis to support effective treatment and long-term care. Chest radiography remains the most widely used imaging modality for pediatric pulmonary assessment. Consequently, reliable AI-assisted diagnostic methods are essential for alleviating the workload of clinical radiologists. However, most existing deep learning-based approaches are data-driven and formulate diagnosis as a black-box image classification task, resulting in limited interpretability and reduced clinical trustworthiness. To address these challenges, we propose a trustworthy two-stage diagnostic paradigm for pediatric chest X-ray diagnosis that closely aligns with the radiological workflow in clinical practice, in which the diagnosis procedure is constrained by evidence. In the first stage, a vision–language model fine-tuned on pediatric data identifies radiological findings from chest radiographs, producing structured and interpretable diagnostic evidence. In the second stage, a multimodal large language model integrates the radiograph, extracted findings, patient demographic information, and external medical domain knowledge with RAG mechanism to generate the final diagnosis. Experiments conducted on the VinDr-PCXR dataset demonstrate that our method achieves 90.1% diagnostic accuracy, 70.9% F1-score, and 82.5% AUC, representing up to a 13.1% increase in diagnosis accuracy over the state-of-the-art baselines. These results validate the effectiveness of combining multimodal reasoning with explicit medical evidence and domain knowledge, and indicate the strong potential of the proposed approach for trustworthy pediatric radiology diagnosis.

## 1. Introduction

Several studies have demonstrated that pediatric respiratory diseases are among the leading causes of hospital admissions, emergency department visits, and childhood mortality worldwide [1]. In particular, acute lower respiratory infections represent the primary cause of mortality in children under five years of age, accounting for approximately 20% of global childhood deaths [2,3]. In U.S. children’s hospitals, respiratory diseases are also among the most prevalent and costly conditions, comprising nearly one-third of all pediatric inpatient admissions [4]. Beyond their high morbidity and mortality, pediatric respiratory diseases can result in long-term health consequences that adversely affect growth, pulmonary function, and overall quality of children’s lives. In China, researchers have been paying attention to the cases displaying heightened severity and refractoriness [5]. Therefore, accurate and timely diagnosis is essential for guiding appropriate clinical management, optimizing follow-up treatment, and improving long-term outcomes.

Rapid technological advances, particularly in artificial intelligence (AI) for healthcare, have reshaped the landscape of medical imaging and enhanced its contribution to disease diagnosis, treatment planning, clinical decision-making and so on. As a cornerstone of routine clinical workflows, radiologic imaging provides critical anatomical and pathological information that supports both initial diagnosis and longitudinal disease monitoring. Among various imaging techniques, chest radiography (CXR) remains the most widely used and clinically indispensable tool for the evaluation of pulmonary diseases due to its low cost, rapid acquisition, minimal radiation exposure, and broad accessibility across healthcare systems worldwide [6]. Consequently, CXR continues to play a central role in frontline screening, disease triage, and follow-up assessment in both adult and pediatric populations. Meanwhile, the interpretation and analysis of chest radiographs have been increasingly influenced by recent progress in artificial intelligence, especially deep learning-based approaches. These methods enable automated extraction of imaging features and have demonstrated the potential to assist radiologists in multiple clinical tasks, including diagnostic decision support and radiology report generation. By supporting clinical decision-making and reducing manual workload, AI-assisted CXR analysis systems offer a promising avenue for improving diagnostic efficiency and consistency. From both a radiological and societal point of view [7,8], the increasing workload for radiologists, the shortage of physicians with complete and standardized training, as well as the uneven distribution of medical resources across regions [9,10], which was extremely obvious during the coronavirus disease 2019 (COVID-19) [11] could be mitigated by the assistance of artificial intelligence.

In recent years, a large number of studies have proposed diverse artificial intelligence-based approaches for radiographic analysis, applying them on different kinds of downstream tasks including disease diagnosis, lesion segmentation, and grounding [12,13,14,15,16]. Most of these methods are predominantly data-driven, relying on deep learning architectures such as convolutional neural networks (CNNs) [17], autoencoders [18], and their variants. While these models have demonstrated promising performance, they typically require large-scale, well-annotated training datasets to achieve robust generalization and reliable predictive accuracy. In the context of pediatric respiratory disease diagnosis, however, the availability of high-quality annotated imaging data is often limited. As a result, the development and deployment of AI-driven radiological solutions with dependable and consistent performance in pediatric populations lag behind those designed for adult patients [19]. This data scarcity not only constrains model training but also exacerbates performance instability and generalization issues when applying existing methods to pediatric clinical scenarios. Moreover, formulating disease diagnosis solely as an image classification task is inherently insufficient and potentially unreliable. Traditional deep learning models generally operate as black-box systems, providing limited interpretability and lacking explicit diagnostic evidence to support their predictions, which reduces clinicians’ confidence in using them. Additionally, in clinical practice, physicians typically integrate imaging findings with information from multiple modalities before reaching a diagnostic conclusion [20]. The absence of transparent reasoning and clinically interpretable evidence in current AI models therefore poses a significant barrier to their acceptance and effective integration into real-world pediatric diagnostic workflows.

Recently, a growing research has explored multimodal large language model (MLLM)-based approaches that flexibly integrate information in images, text, and other modalities. Representative models such as LLaVA [21] and BLIP-2 [22], along with commercial MLLMs including GPT-4 [23] and Gemini [24], have demonstrated strong performance across a wide range of downstream tasks. By jointly modeling visual and language representations, these models provide new opportunities for unified reasoning over heterogeneous data sources. Despite these, the application of MLLM-based paradigms to medical imaging remains limited, particularly in radiology and pediatric diagnostic scenarios. Medical image interpretation relies heavily on fine-grained visual features, domain-specific knowledge, and the accumulated clinical experience of physicians, which are not fully captured by general MLLMs. Consequently, only a limited number of studies have specifically investigated MLLM-driven frameworks tailored to medical or pediatric radiology diagnosis. Moreover, recent approaches such as chain-of-thought supervised models and reinforcement learning-enhanced frameworks mainly focus on improving reasoning fluency or report generation quality [25,26,27]. Multi-agent systems further attempt to simulate expert collaboration through modular orchestration [28]. However, these methods typically operate in an end-to-end or loosely structured fashion, without explicitly disentangling perception from diagnostic reasoning or aligning the model architecture with real-world clinical workflows.

To address these limitations, we propose a two-stage, trustworthy framework for pediatric radiology diagnosis that is explicitly designed to align with authentic clinical radiology workflows. In the first stage, vision–language pretrained models fine-tuned on pediatric data act as radiologists by identifying and recognizing abnormal findings in chest radiographs. These suspected findings are then used as interpretable evidence and provided to an MLLM, which functions as a specialist physician to support precise diagnostic reasoning in the second stage. During the diagnostic process, authoritative clinical guidelines, disease descriptions, and expert consensus are incorporated to enhance domain-specific medical knowledge. This information is retrieved and utilized through a retrieval-augmented generation (RAG) mechanism, enabling the MLLM to ground its diagnostic decisions in clinically reliable references.

In summary, the main contributions of this work are as follows:

Trustworthy pediatric radiology diagnostic framework.

We propose a two-stage framework that is consistent with radiological workflows in clinical practice, making the diagnostic results more credible and interpretable for clinical reference.

Knowledge-enhanced diagnosis based on MLLMs. In the proposed framwork, the final disease diagnosis process is enhanced through the integration of medical domain knowledge.

State-of-the-art performance on VinDr-PCXR [29]. The proposed method achieves state-of-the-art performance across all evaluation metrics on the test set of the largest publicly available pediatric chest X-ray dataset.

## 2. Method

**Figure 1:**
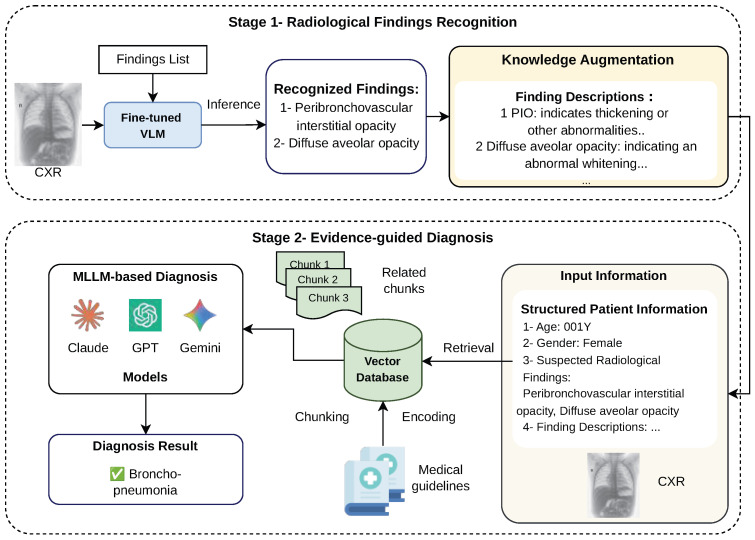
Overview of the proposed evidence-guided two-stage diagnostic reasoning framework for pediatric chest radiology.

The overall diagnostic framework is designed to closely align with the standard radiological workflow in clinical practice. The proposed two-stage framework is illustrated in Figure 1. In routine clinical settings, following image acquisition by radiology technicians, radiologists interpret chest radiographs by identifying abnormal radiological findings and generating structured reports. These findings serve as critical references for specialist physicians, such as pediatricians, who further integrate imaging evidence with clinical information to establish an accurate diagnosis and formulate an appropriate treatment plan. Motivated by this established workflow, we design a two-stage diagnostic framework that explicitly separates radiological finding recognition from disease-level diagnostic reasoning, thereby improving explainability and traceability. In the first stage, a vision–language model (VLM) fine-tuned on pediatric radiological data interprets the input chest X-ray and identifies abnormal radiological findings. The recognized findings are then organized into structured and interpretable diagnostic evidence, which serves as the input to the second stage of the framework. In the second stage, a multimodal large language model (MLLM) performs disease diagnosis by reasoning over the evidence generated in the first stage. To compensate for the limited medical domain knowledge of general-purpose MLLMs, additional domain-specific information, including standardized descriptions of radiological findings and patient demographic attributes, is incorporated into the diagnostic process. Specifically, the structured findings, demographic information, and the chest radiograph jointly constitute the input query to the MLLM. Relevant medical knowledge is retrieved from an external database via a retrieval-augmented generation (RAG) mechanism. Finally, the diagnostic MLLM integrates multimodal information from imaging evidence, retrieved medical knowledge, and structured patient data to produce the final disease diagnosis. The following sections describe each module of the proposed framework in detail.

### 2.1. Vision-LanguageModel Domain-Specific Fine-Tuning

Radiological findings in chest radiographs are often localized within small anatomical regions and manifested through subtle, fine-grained visual patterns, such as faint opacities, mild consolidations, or slight structural asymmetries. These characteristics make precise recognition particularly challenging, especially in pediatric chest X-rays, where anatomical variations across age groups further increase diagnostic complexity. In this stage, vision–language models (VLMs) are employed to perform radiological finding recognition, leveraging their capability to jointly model visual representations and textual semantics for fine-grained medical interpretation.

Benefiting from the availability of large-scale adult multimodal chest X-ray datasets, including MIMIC-CXR [30], VinDr-CXR [31], and CheXpert [32], VLMs pretrained on these resources have acquired rich radiological representations and substantial medical domain knowledge. Through large-scale pretraining, these models are able to capture clinically meaningful visual patterns and learn robust semantic alignments between chest radiographs and radiological descriptions. Such properties make pretrained VLMs particularly suitable for transfer learning to downstream radiological tasks under limited data settings.

In this work, we adopt the knowledge-enhanced auto diagnosis (KAD) model [33] as the initialization backbone for fine-tuning. Unlike general-purpose vision–language models such as LLaVA or BLIP-2, which are pretrained mainly on natural image–text pairs without explicit medical supervision, KAD is jointly pretrained on adult chest radiographs, corresponding radiology reports, and structured medical knowledge graphs. By leveraging both visual representations and medical knowledge embeddings learned from large-scale adult chest radiography datasets, KAD provides a strong and clinically meaningful starting point for pediatric radiological adaptation. Given the limited availability of publicly accessible pediatric multimodal pretrained models, transferring knowledge from adult-domain radiology data becomes a practical and effective strategy. The semantic associations between imaging findings and disease concepts learned by KAD during adult pretraining can be effectively adapted to pediatric scenarios through domain-specific fine-tuning, thereby alleviating the cold-start problem caused by pediatric data scarcity and improving radiological finding recognition robustness.

**Figure 2:**
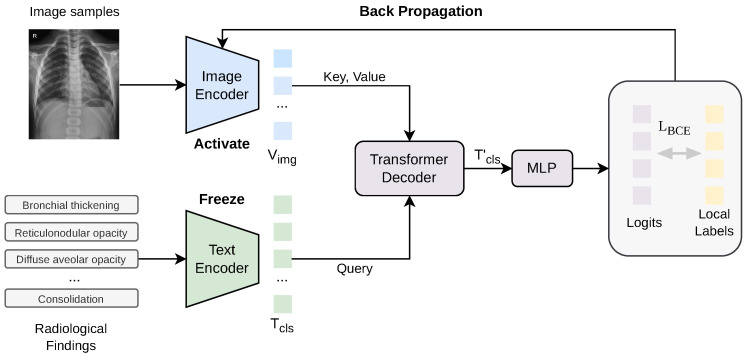
Fine-tuning procedure of the visual–language model for radiological findings recognition.

The overall fine-tuning process is illustrated in Figure 2, which is a domain adaptation process. By leveraging both visual representations and medical knowledge embeddings learned from adult data, KAD provides a strong and informative starting point for pediatric radiological adaptation. Specifically, the KAD model consists of an image encoder and a knowledge encoder (text encoder), both of which are pretrained on paired adult chest X-ray images and associated radiological reports. In our framework, the pretrained weights of these two encoders are used to initialize the VLM, which is subsequently fine-tuned using pediatric chest X-ray data. During fine-tuning, the model adapts the learned adult radiological representations to pediatric imaging characteristics while preserving clinically relevant semantic associations. This transfer learning strategy facilitates the effective migration of adult radiological knowledge to the pediatric domain, alleviates the cold-start problem, and improves radiological finding recognition performance under limited pediatric data availability.

After extracting visual and textual representations, the KAD architecture employs a transformer decoder to fuse multimodal information and model the interactions between image features and semantic concepts. Specifically, let $V_{\mathrm{img}}$ denote the visual patch features extracted from the input chest X-ray image, and let $T_{\mathrm{cls}}$ represent the class-specific textual embeddings corresponding to radiological findings. The image encoder $E_{\mathrm{img}}$ and text encoder $E_{\mathrm{txt}}$ are used to map the input modalities into a shared embedding space.

Formally, given an input chest radiograph *X*, the visual features are obtained through the image encoder as

(1) $$V_{\mathrm{img}}=E_{\mathrm{img}}\left(X\right)\in R^{N\times d},$$

where *N* denotes the number of visual patches and *d* represents the feature dimension.

Similarly, given a predefined set of radiological finding categories C, the corresponding textual embeddings are generated by the text encoder as

(2) $$T_{\mathrm{cls}}=E_{\mathrm{txt}}\left(C\right)\in R^{|C|\times d},$$

where |C| denotes the number of predefined radiological finding classes and *d* is the embedding dimension. The resulting visual features $V_{\mathrm{img}}$ and textual embeddings $T_{\mathrm{cls}}$ are subsequently fed into a transformer-based decoder, which performs multimodal fusion through cross-attention mechanisms to model fine-grained interactions between localized image regions and semantic radiological concepts, thereby enabling accurate radiological finding recognition.

Within the transformer-based decoder, a cross-attention mechanism is employed to enable each class-specific textual feature $T_{\mathrm{cls}}^{\left(i\right)}$ to attend to the visual feature space extracted from the input chest radiograph. Through this mechanism, each radiological finding class actively queries image regions that are most relevant to its corresponding semantic representation, allowing the model to establish fine-grained associations between localized visual evidence and predefined clinical concepts.

Formally, the attention weight distribution $\alpha_{i}$ for the *i*-th radiological finding class is computed as

(3) $$\alpha_{i}=\mathrm{Softmax}\;\left(\frac{T_{\mathrm{cls}}^{\left(i\right)}W_{q}{\left(V_{\mathrm{img}}W_{k}\right)}^{⊤}}{\sqrt{d}}\right),$$

where $W_{q}$ and $W_{k}$ denote the learnable projection matrices for the query and key in the transformer mechanism, respectively, and *d* represents the dimensionality of the feature embeddings. The resulting attention weights highlight image regions that contribute most strongly to the prediction of the corresponding radiological finding.

By explicitly modeling the attention weight distribution between class-specific textual features and visual features, the transformer decoder is able to dynamically identify the most informative image patches that provide strong evidence for the presence of a given radiological finding. The attended visual information is then aggregated and fused into the corresponding class representation. Specifically, the fused class-aware feature ${T^{\prime }}_{\mathrm{cls}}^{\left(i\right)}$ is computed as

(4) $${T^{\prime }}_{\mathrm{cls}}^{\left(i\right)}=\alpha_{i}\left(V_{\mathrm{img}}W_{v}\right)+T_{\mathrm{cls}}^{\left(i\right)},$$

where $W_{v}$ denotes the learnable value projection matrix. This residual fusion mechanism preserves the original semantic prior encoded in each class embedding while augmenting it with image-specific visual evidence. As a result, the model effectively integrates multimodal information, enabling more accurate and interpretable recognition of radiological findings.

This formulation explicitly illustrates how each class token selectively aggregates image evidence that supports the presence of the corresponding radiological finding. By establishing class-aware interactions between textual representations and visual features, the model effectively associates specific radiological findings with their most relevant image regions.

To transform the high-dimensional fused class features into final classification prediction scores (i.e., logits), additional feature aggregation and projection operations are applied. Specifically, the fused class-aware features $T_{\mathrm{cls}}^{\prime }\in R^{C\times d}$ are first aggregated via average pooling along the category dimension, yielding a compact global representation:

(5) $$\bar{T}=\frac{1}{C}\sum_{i=1}^{C}{T^{\prime }}_{\mathrm{cls}}^{\left(i\right)}\in R^{d}.$$

Subsequently, the aggregated feature $\bar{T}$ is fed into a linear classification head implemented as a multi-layer perceptron (MLP), which projects the representation from the embedding space of dimension *d* to the target class space of dimension *C*:

(6) $$z=\mathrm{MLP}\left(\bar{T}\right)\in R^{C},$$

where *z* denotes the final prediction logits for multi-label radiological finding classification.

The radiological finding recognition task is formulated as a multi-label classification problem, in which multiple pathological findings may simultaneously exist within a single chest radiograph. Accordingly, the binary cross-entropy (BCE) loss is employed as the training objective to supervise the model optimization, as defined in Equation (7). The fine-tuning process aims to minimize this loss, which quantitatively measures the discrepancy between the predicted logits and the corresponding ground-truth labels through gradient-based optimization.

During training, gradients are computed via backpropagation and propagated backward along the forward computation graph. As illustrated in Figure 2, the text encoder is kept frozen to preserve the pretrained semantic representations learned during the pretraining stage, while the image encoder and subsequent multi-modal fusion modules are jointly optimized. Specifically, the gradient flow originates from the loss layer, propagates through the linear classification head and the cross-attention layers of the Transformer decoder, and finally reaches the convolutional and projection layers of the image encoder. This selective fine-tuning strategy enables the model to effectively adapt visual representations to pediatric-specific radiological patterns while maintaining stable and consistent textual embeddings for radiological findings. Such a design helps mitigate overfitting and preserves the semantic alignment between visual features and medical concepts learned from large-scale adult datasets. All trainable parameters are optimized using the AdamW optimizer, which combines adaptive learning rates with decoupled weight decay, thereby improving both training stability and generalization performance.

(7) $$L=-\frac{1}{B\cdot C}\sum_{b=1}^{B}\sum_{i=1}^{C}\left[y_{b,i}\log\left(\sigma \left(z_{b,i}\right)\right)+\left(1-y_{b,i}\right)\log\left(1-\sigma \left(z_{b,i}\right)\right)\right],$$

where

- L denotes the binary cross-entropy loss;
- *B* represents the batch size, and *C* denotes the number of predefined radiological finding classes;
- $z_{b,i}$ is the predicted logit for the *i*-th class of the *b*-th sample;
- $y_{b,i}\in \left\{0,1\right\}$ is the corresponding ground-truth label;
- σ(·) denotes the sigmoid activation function.

### 2.2. Domain Knowledge-Enhanced Multimodal Diagnosis

In the second stage of the proposed framework, multimodal large language models (MLLMs) are employed to act as specialist physicians and perform disease-level diagnostic reasoning tasks. At this stage, the MLLM conducts diagnostic reasoning by comprehensively referring to multimodal input information, including the chest radiograph, the recognized radiological findings obtained from Stage 1, and external medical domain knowledge retrieved from an authoritative medical knowledge vector database.

By integrating these complementary sources of information, the MLLM is able to emulate the diagnostic reasoning process of experienced clinicians, who do not rely solely on imaging evidence but also incorporate accumulated medical knowledge and patient-specific contextual information when formulating a diagnosis. Moreover, the inclusion of retrieved domain knowledge explicitly grounds the model’s reasoning in established clinical guidelines and expert consensus, which enhances diagnostic reliability and robustness while effectively mitigating the risk of hallucinated or unsupported conclusions.

The Stage 2 diagnostic module consists of two main components. The first component is responsible for integrating heterogeneous input information into a unified, structured, and clinically meaningful representation. This process ensures that visual evidence from chest radiographs, structured radiological findings recognized in Stage 1, and patient demographic information are coherently organized and jointly presented to the model in a consistent format. Such structured integration facilitates effective cross-modal interaction and provides a comprehensive diagnostic context. The second component performs disease-level diagnostic reasoning enhanced by clinical domain knowledge. In this component, the MLLM leverages relevant medical knowledge retrieved from authoritative clinical guidelines and disease descriptions to guide its reasoning process. By grounding the diagnostic inference in external domain knowledge, the model is able to generate accurate, reliable, and evidence-supported diagnostic conclusions, thereby improving clinical interpretability and reducing the risk of unsupported or hallucinated predictions.

#### 2.2.1. Structured Input Integration

Following the recognition stage, the identified radiological findings are treated as explicit diagnostic evidence for subsequent disease inference. To address the limited medical domain knowledge of general MLLMs—particularly in the specialized context of pediatric radiology, we supplement each recognized finding with concise and standardized clinical descriptions. These descriptions are collected from authoritative medical resources and databases, such as Radiopaedia and RSNA, and are further reviewed and validated by clinical experts to ensure their accuracy and reliability.

By incorporating expert-curated textual descriptions alongside the recognized findings, the multimodal input is transformed into a structured and clinically meaningful representation. This structured integration enables the MLLM to better interpret the clinical significance of each finding, bridge knowledge gaps between visual observations and medical concepts, and establish a stronger foundation for downstream knowledge-augmented diagnostic reasoning.

The demographic information, including gender and age, is also important for the clinicians to consider when diagnosing the pediatric respiratory disease, according to the authoritative medical references [11]. Therefore, the demographic information extracted from the original DICOM files is taken into consideration as part of the patient information.

In summary, the structured input information for each patient sample is composed of multiple clinically relevant components. Specifically, it includes:

- Gender, encoded as a categorical variable (”M” or ”F”).
- Age, which provides essential demographic context for pediatric diagnosis.
- Recognized radiological findings, automatically identified in Stage 1 and serving as the primary visual evidence.
- Clinical descriptions of the recognized findings, collected from authoritative medical sources and validated by experts to provide domain knowledge support.

Together, these elements form a structured and comprehensive representation of each patient sample, enabling the multimodal large language model to reason over both demographic information and knowledge-enhanced radiological evidence during the diagnostic process.

#### 2.2.2. Retrieval-Augmented Diagnosis

The final diagnosis is performed by a multimodal large language model (MLLM), which integrates heterogeneous sources of evidence, including structured patient information introduced in the previous section, the original chest X-ray, and external medical domain knowledge retrieved from a curated knowledge base. By jointly reasoning over visual, textual, and knowledge-based inputs, the MLLM is able to produce accurate and clinically trustworthy diagnostic results. The overall diagnostic workflow of the second stage is illustrated in the lower part of Figure 1.

Knowledge Source and Construction. In clinical practice, diagnostic decisions are primarily guided by authoritative clinical guidelines and expert consensus documents. Accordingly, we construct the external knowledge base using high-quality resources from reputable medical platforms, including Radiopaedia, the Radiological Society of North America (RSNA), Pediatric Imaging: A Pediatric Radiology Textbook, and the Pediatric Radiology Digital Library. These sources are widely recognized and routinely consulted by radiologists and clinicians in pediatric settings. From these references, we specifically extract the imaging- and radiology-related sections that describe characteristic radiographic manifestations of different pediatric respiratory diseases. These sections focus on disease-specific imaging features, typical radiological patterns, and differential diagnostic clues observable on chest X-rays.

Maintenance and Updating Strategy. To ensure long-term sustainability, the knowledge base follows a version-controlled update protocol. Newly published guidelines or consensus documents collected are periodically reviewed and incorporated after clinical verification. Each knowledge entry is associated with metadata (source, publication year, version ID), enabling traceability and reproducibility across different system versions.

Validation and Clinical Reliability. To ensure the reliability and clinical validity of the knowledge base, as well as consistency with established clinical standards, all collected content is carefully reviewed and verified by pediatric specialists.

For efficient retrieval and structured utilization by the MLLM, the curated knowledge is organized into predefined templates, with each disease guideline stored as an independent Markdown document. This structured representation facilitates accurate knowledge retrieval and seamless integration into the retrieval-augmented diagnostic process, enabling the MLLM to ground its diagnostic reasoning in authoritative clinical evidence. The textual content is then encoded into vector embeddings using a text encoder $f_{\mathrm{enc}}$. The textual content of each guideline is further encoded into a dense vector representation using a text encoder $f_{\mathrm{enc}}$. For a given disease guideline $K_{j}$, its embedding is computed as

(8) $$kj=f\mathrm{enc}(K_{j}),\;j=1,2,\ldots ,N,$$

where $k_{j}\in R^{d}$ denotes the encoded knowledge vector in the shared embedding space, and *N* represents the total number of diseases included in the knowledge base. All knowledge embeddings are subsequently indexed in a vector database to support efficient similarity-based retrieval.

During the retrieval stage, the MLLM constructs a query vector *q* by jointly considering the patient demographic information, the radiological findings recognized in Stage 1, and its internal reasoning context. The relevance between the query and each disease guideline embedding is measured using cosine similarity:

(9) $$S\left(q,k_{j}\right)=\frac{q\cdot k_{j}}{\left|q|,|kj\right|},$$

Based on the similarity scores, the most relevant disease guideline is selected as

(10) $$K^{*}=\underset{j}{arg max}S\left(q,k_{j}\right),$$

which is then injected into the MLLM as external knowledge to augment the final diagnostic reasoning process.

The final diagnostic prediction is produced by jointly processing the input chest radiograph *i*, the recognized radiological findings RS, patient demographic information DI, the predefined system role *r*, the task specification *T*, and the retrieved clinical guideline $K^{*}$. This process can be formally expressed as

(11) $$OR=\mathrm{MLLM}(i,RS,DI,r,T,K^{*}),$$

where OR denotes the final diagnostic output result corresponding to the predicted disease category. By integrating multimodal patient-specific evidence with retrieval-augmented authoritative medical knowledge, the MLLM is guided to perform clinically grounded reasoning, thereby improving diagnostic reliability, consistency, and interpretability in pediatric diagnosis scenarios.

For completeness and improved transparency, an algorithmic summary of the proposed diagnostic framework is provided below. Algorithm 1 formalizes the entire pipeline using the notation defined above and clarifies the interactions among feature extraction, multimodal representation learning, knowledge retrieval, and final inference of the two stages. This structured presentation facilitates both conceptual understanding and practical implementation.
**Algorithm 1** Retrieval-Augmented Multimodal Diagnostic Framework.**Require:** Chest radiograph *X*; radiological finding categories C; knowledge base ${\left\{K_{j}\right\}}_{j=1}^{N}$; demographic information DI; system role *r*; task *T***Ensure:** Final diagnostic output OR  1:// Stage 1: Radiological Finding Recognition  2:$V_{\mathrm{img}}=E_{\mathrm{img}}\left(X\right)$  3:$T_{\mathrm{cls}}=E_{\mathrm{txt}}\left(C\right)$  4:Compute cross-attention between $T_{\mathrm{cls}}$ and $V_{\mathrm{img}}$  5:Obtain fused features $T_{\mathrm{cls}}^{\prime }$  6:Aggregate $\bar{T}=\frac{1}{C}\sum_{i=1}^{C}{T^{\prime }}_{\mathrm{cls}}^{\left(i\right)}$  7:$z=\mathrm{MLP}(\bar{T})$  8:Derive recognized findings RS from z  9:// Stage 2: Evidence-guided Diagnosis10:**for** j=1,…,N **do**11:      $k_{j}=f_{\mathrm{enc}}\left(K_{j}\right)$12:**end for**13:Construct query *q* using DI and RS14:$K^{*}={arg max}_{j}S\left(q,k_{j}\right)$15:$OR=\mathrm{MLLM}(X,RS,DI,r,T,K^{*})$16:**return** OR

## 3. Experiments

### 3.1. Dataset

The dataset used for model training and evaluation is derived from the VinDr-PCXR dataset [29], which comprises 9125 pediatric chest radiograph studies collected at a large tertiary pediatric hospital in Vietnam between 2020 and 2021. The dataset covers a wide range of pediatric age groups and respiratory conditions, providing a representative benchmark for pediatric chest X-ray analysis. All radiographic images were independently annotated by experienced, board-certified radiologists in accordance with standardized clinical annotation protocols. This rigorous annotation process ensures high-quality and reliable ground-truth labels for both radiological findings and disease-level diagnoses. A statistical summary of the dataset, including the distribution of diagnostic categories and demographic information, is presented in Table 1.

The original data in the dataset are stored in the Digital Imaging and Communications in Medicine (DICOM) format. For the convenience of image processing and visualization, all radiographs are first converted into PNG format, during which patient demographic information embedded in the DICOM metadata is also extracted. Prior to model training, all chest X-ray images are resized to a unified spatial resolution of 512×512 pixels. This pre-processing step ensures consistent input dimensionality across all samples, which is critical for stable optimization and effective feature extraction in deep neural networks. The VinDr-PCXR dataset provides two levels of expert annotations: local labels and global labels. The local labels describe fine-grained radiological findings at the image level, such as reticulonodular opacity and bronchial wall thickening, enabling detailed characterization of localized visual abnormalities. In contrast, the global labels correspond to study-level diagnostic categories that reflect the overall clinical interpretation of each case. In this work, the global labels are adopted as the ground truth for evaluating disease-level diagnostic performance, while the local labels are leveraged to support radiological finding recognition and structured evidence generation.

In total, the dataset contains 37 local radiological finding categories, including the ”No finding” label, and 17 global disease categories. To ensure reliable evaluation and mitigate the adverse effects of severe class imbalance, disease categories with extremely limited sample sizes (fewer than five positive cases) are merged into a unified “Other diseases” category, following the common practice adopted in prior studies [34,35,36,37]. After category consolidation, the final disease-level classification task consists of six diagnostic categories: “No finding” (907 samples), “Bronchitis” (174 samples), “Broncho-pneumonia” (84 samples), “Bronchiolitis” (90 samples), “Pneumonia” (89 samples), and “Other diseases” (81 samples). This consolidation strategy achieves a balance between preserving clinically meaningful diagnostic distinctions and ensuring sufficient statistical robustness for reliable model training and evaluation.

**Figure 3:**
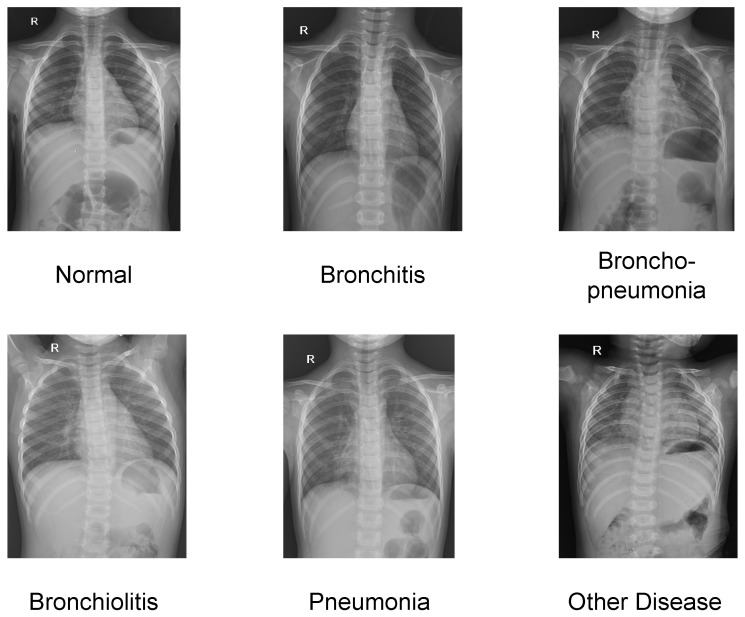
Representative pediatric chest X-ray samples from the VinDr-PCXR dataset, covering six diagnostic categories.

Figure 3 presents representative chest radiographs from each diagnostic category, illustrating the diversity of imaging appearances across different disease types. All experimental results reported in this study are obtained using the official test split of the VinDr-PCXR dataset to ensure fair and reproducible evaluation.

### 3.2. Fine-Tuning Implementation

The image encoder adopts a ResNet-50 architecture, while BERT is employed as the text encoder. Both encoders are initialized with publicly available pretrained weights obtained from the training on public adult radiology dataset rather than being trained from scratch, enabling effective knowledge transfer and faster convergence during fine-tuning. The input to the image encoder is a chest radiograph resized to 512×512×3, where the three channels correspond to the replicated grayscale image. After feature extraction, the visual feature map is projected through two multilayer perceptron (MLP) layers to obtain patch embeddings with a dimensionality of 768, which is aligned with the hidden size of the text encoder to facilitate multimodal feature fusion.

All fine-tuning experiments are implemented using the PyTorch 2.6.0 deep learning framework and conducted on NVIDIA GeForce RTX 3090 GPUs with 24 GB of memory. Model optimization is performed using the AdamW optimizer. The initial learning rate is set to $2\times 10^{-5}$, which is empirically chosen to balance convergence speed and training stability. The model is trained for a total of 80 epochs with a batch size of 32. This configuration ensures sufficient training iterations while maintaining computational efficiency and stable gradient updates throughout the fine-tuning process.

For the evaluation of the radiological finding recognition stage, several standard classification metrics are employed to comprehensively assess model performance, including accuracy (ACC), precision, recall, and F1-score. These metrics provide complementary perspectives on the model’s predictive capability for diagnosis task, enabling a balanced evaluation of both overall performance and class-specific behavior.

Accuracy (ACC) reflects the proportion of correctly predicted labels over all predictions, serving as a global measure of classification correctness. Precision quantifies the reliability of positive predictions by measuring the fraction of true positive findings among all predicted positives, while recall evaluates the model’s ability to identify relevant radiological findings by measuring the proportion of true positives among all ground-truth positives. The F1-score, defined as the harmonic mean of precision and recall, provides a single summary metric that balances false positives and false negatives, which is particularly important in medical image analysis where both types of errors can have significant clinical implications.

### 3.3. MLLM-Based Diagnosis Implementation

In our experiments, we evaluate three representative multimodal large language models (MLLMs) that have demonstrated strong and reliable performance on a wide range of multimodal reasoning benchmarks, namely GPT-4o, Gemini-2.5 Pro, and Claude-3.5 Sonnet. These models are selected due to their advanced capabilities in jointly processing visual and textual inputs, as well as their proven effectiveness in complex reasoning tasks involving heterogeneous information sources. For the retrieval-augmented generation (RAG) component, the text embedding model text-embedding-3-small provided by OpenAI is utilized to encode external clinical guidelines and disease-related knowledge into dense vector representations. By combining strong multimodal reasoning models with a dedicated medical knowledge retrieval mechanism, the proposed framework aims to ensure both diagnostic accuracy and knowledge-grounded inference.

The diagnostic prompt is carefully designed and organized into five key components: (1) **Role**, (2) **Disease List**, (3) **Task**, (4) **Decision Rule**, and (5) **Output Format**. The Disease List explicitly defines the set of candidate diagnostic categories, thereby constraining the model’s output space and reducing ambiguity in the generated predictions. This explicit restriction is particularly important for clinical diagnosis, where uncontrolled generation may lead to unsupported or irrelevant disease labels. The Role component specifies the clinical identity assumed by the MLLM, namely a pediatric physician with expertise in respiratory diseases. By assigning a well-defined clinical role, the prompt encourages the model to adopt a professional diagnostic perspective and align its reasoning process with real-world clinical practice. The Task and Decision Rule components jointly define the diagnostic objective and provide additional constraints and guidance, promoting clinically consistent, evidence-based, and logically structured reasoning. Finally, the Output Format enforces a standardized disease name as the model output. This standardized formatting ensures uniformity across predictions, facilitates reliable parsing and comparison of results, and enables robust quantitative evaluation across different MLLMs under consistent experimental settings.

Here shows the system prompt used in the diagnosis experiment:

**# Role:** You are an experienced respiratory physician specializing in interpreting chest radiographs with high diagnostic accuracy.

**# Disease List:** "Bronchitis"; "Broncho-pneumonia"; "Bronchiolitis"; "Pneumonia"; "Other disease"; "No finding".

**# Task:** Interpret: Based on the radiograph and patient information, determine the most accurate diagnosis from the allowed list. Verify findings: Compare the provided radiology findings with the radiograph.

If the provided findings say “No finding”, you must carefully re-check the radiograph and not rely solely on the provided label.

**Decision Rule:** If none of the listed diseases fits, output "Other disease". If the patient is normal, output "No finding".

**# Output Format:** Diagnosis result: one or more of the six allowed diagnoses.

Patient information is provided to the MLLM in the form of a user prompt, which includes patient demographic details (age and sex), as well as the suspected radiological findings identified in Stage 1 together with their corresponding professional explanations and standardized clinical descriptions.

The integrated patient information is subsequently formulated as a structured query and submitted to a medical knowledge vector database constructed using Milvus. Both the query and the curated external medical knowledge are embedded into dense vector representations using the text-embedding-3-small model. During the knowledge retrieval process, cosine similarity is computed between the query embedding and all knowledge entries stored in the vector database. We empirically evaluated different Top-k retrieval settings (k = 1–5) and selected the Top-3 configuration based on validation performance (see Section 4). These retrieved guideline sections, which are most relevant to the patient’s imaging findings and demographic context, are then incorporated into the retrieval-augmented generation (RAG) pipeline and explicitly referenced during the final diagnostic reasoning performed by the MLLM.

For the diagnostic stage, we conduct a comprehensive evaluation of three state-of-the-art multimodal large language models (MLLMs), selected based on their demonstrated capabilities in multimodal reasoning, performance on medical-related tasks, and overall cost-effectiveness in practical applications. Specifically, the models chosen are GPT-4o (OpenAI), Claude-3.5 Sonnet (Anthropic), and Gemini-2.5 Pro (Google). Each of these MLLMs is designed to integrate multiple sources of information—including detailed multimodal patient data, externally retrieved medical knowledge, and the corresponding chest radiographs—to generate a coherent and clinically relevant diagnostic prediction. This integration enables the models to leverage both structured and unstructured medical knowledge, thereby enhancing their potential for accurate and evidence-aware reasoning in complex diagnostic scenarios.

To evaluate the diagnostic performance of these models, we employ a series of standard classification metrics, including accuracy, precision, recall, and F1-score, which collectively provide a quantitative assessment of prediction correctness, reliability, and balance between sensitivity and specificity. Furthermore, the area under the receiver operating characteristic curve (AUC) is calculated to capture the models’ overall discriminative capability. By incorporating AUC, we provide a threshold-independent measure of diagnostic robustness, enabling a more comprehensive understanding of each model’s ability to distinguish between different clinical outcomes under varying conditions.

## 4. Results

### 4.1. Radiological Finding Recognition Performance

We first evaluate the performance of Stage 1, namely the radiological finding recognition task, by comparing the results on the VinDr-PCXR test set before and after fine-tuning on the corresponding training set. In this stage, the evaluation is conducted using the local annotations in the dataset, which correspond to 36 fine-grained radiological findings. To comprehensively assess model performance, five commonly used diagnostic metrics are adopted as mentioned before, including accuracy, recall, precision, F1-score, and the area under the receiver operating characteristic curve (AUC).

The quantitative comparison results are summarized in Table 2. To provide a thorough benchmarking of model performance on the VinDr-PCXR test set, we evaluate several representative approaches—(1) a baseline ViT-B/16 model trained from scratch on the VinDr-PCXR training set; (2) GPT-4o applied with a direct recognition prompt; (3) the knowledge-enhanced auto diagnosis (KAD) vision–language model without any pediatric-specific fine-tuning; and (4) the KAD model after fine-tuning on the VinDr-PCXR training set—illustrating the performance gains achieved through task-specific adaptation. This comparison enables a clear assessment of the impact of model architecture, knowledge integration, and dataset-specific fine-tuning on radiological finding recognition accuracy and robustness.

As shown in Table 2, the fine-tuned KAD model consistently achieves the best performance across all evaluation metrics. These results demonstrate the effectiveness of transferring adult radiological knowledge to the pediatric domain through targeted fine-tuning, and validate the proposed model selection strategy and training procedure for accurate radiological finding recognition within our framework.

### 4.2. Overall Diagnostic Performance

The overall diagnostic performance of the proposed framework is summarized in Table 3. To ensure a comprehensive evaluation, we compare our method with five representative approaches, encompassing both conventional deep learning models and recent large language model-based frameworks, all assessed on the same VinDr-PCXR test set. All the compared approaches follow the same dataset partition, evaluation protocol, and disease category merging strategy as our work, thereby ensuring the fairness and consistency of the comparison. Methods that did not adopt the same category merging strategy were excluded to avoid potential bias in metric calculation. In addition, we report the results obtained using three different multimodal large language models (MLLMs) within our framework. Among the tested MLLMs, GPT-4o demonstrates the best performance across all evaluation metrics, followed by Claude-3.5 Sonnet in second place. As detailed in Table 3, our framework with GPT-4o achieves the highest accuracy, precision, F1-score, and area under the receiver operating characteristic curve (AUC), with respective values of 0.901, 0.709, 0.709, and 0.835. While the recall score is slightly lower than that achieved by CurriMAE [37], the consistent improvements across the remaining metrics underscore the strong ability and robustness of the proposed approach. These findings suggest that the integration of precise radiological evidence extraction with retrieval-augmented multimodal reasoning substantially enhances diagnostic accuracy for pediatric chest X-rays.

### 4.3. Per-Class Diagnostic Analysis

In addition to overall performance, we further analyze class-wise diagnostic results to better understand and analyze the strengths and limitations of the proposed framework. The per-category performance across different MLLMs is reported in Table 4. As observed, GPT-4o and Gemini-2.5 Pro consistently outperform Claude-3.5 Sonnet across most disease categories and evaluation metrics. This consistent advantage suggests that stronger multimodal reasoning and knowledge integration capabilities contribute to improved diagnostic performance, particularly in complex pediatric radiology scenarios.

### 4.4. Ablation Study

#### 4.4.1. Ablation Study on Framework Components

**Figure 4:**
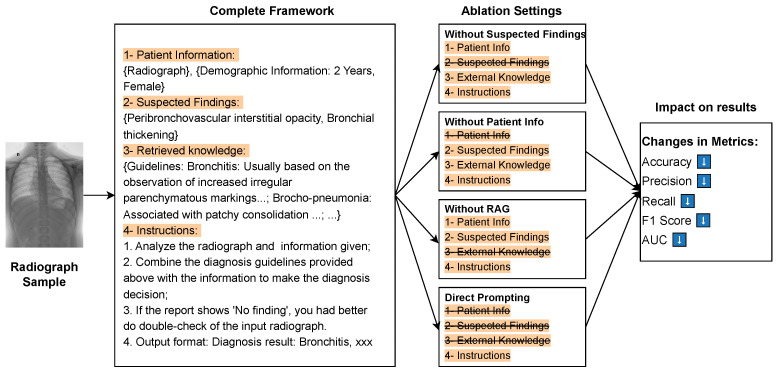
Schematic illustration of the ablation study settings for the proposed evidence-guided diagnostic reasoning framework. The crossed-out parts are the modules that were dissolved during the experiment.

Our proposed framework integrates multiple knowledge sources and configurable MLLM components, making it essential to investigate the individual contribution of each module to the overall performance. To this end, we conduct a comprehensive ablation study using a randomly selected subset of 500 images from the test set, based on GPT-4o as the reasoning model. The experimental configurations of our ablation study are illustrated in Figure 4, while the complete quantitative results are reported in Table 5. Specifically, we evaluate the framework under four distinct experimental settings by systematically removing key components and comparing the resulting performance against the full framework configuration. Three critical modules are ablated in turn: (1) the recognized radiological findings generated in Stage 1, (2) the retrieval-augmented generation (RAG) module, and (3) the patient demographic information input. This ablation design allows us to isolate and quantitatively measure the contribution of each module to overall performance and robustness.

As shown in Table 5, the complete framework incorporating all modules consistently achieves the best performance across all evaluation metrics. In contrast, removing any single component leads to a noticeable degradation in performance, demonstrating that each module plays a non-trivial and complementary role in the diagnostic process. These results underscore the necessity of integrating structured radiological evidence, domain knowledge retrieval, and patient-specific information to achieve reliable and accurate pediatric radiology diagnosis.

#### 4.4.2. Ablation of Retrieval Configuration

In the RAG module of our designed framework, the number of chunks, Top-k, was set as 3 after systematic evaluation across different Top-k settings (k = 1–5) by varying the number of retrieved knowledge chunks incorporated into the diagnostic reasoning process. The quantitative results, summarized in Table 6, were obtained on the same subset used in the ablation study above, with GPT-4o employed to get the results. As shown in the results, diagnostic performance improves gradually when increasing k from 1 to 3. Specifically, the Top-3 configuration achieves the best overall performance across all evaluation metrics, with an accuracy of 0.8775, precision of 0.7845, recall of 0.7790, F1-score of 0.7817, and AUC of 0.5821. However, when further increasing the number of retrieved knowledge chunks to Top-4 and Top-5, performance consistently declines across all metrics. This phenomenon suggests that while incorporating additional knowledge fragments may enrich contextual information, excessive retrieval can introduce weakly related or redundant content, thereby increasing reasoning noise and slightly degrading diagnostic accuracy. Therefore, the results indicate that Top-3 retrieval provides an optimal balance between knowledge sufficiency and information relevance, supporting the selection in the final framework configuration.

#### 4.4.3. Ablation of Probability Integration

Since the proposed method is designed as a two-stage framework, it is necessary to examine the potential impact of Stage 1 recognition errors on Stage 2 diagnostic reasoning. Aimed at this, we conduct an ablation study on evidence representation to investigate whether uncertainty information can mitigate error propagation in the cascaded framework. In the original design, Stage 1 outputs predicted radiological findings, and only those exceeding a predefined confidence threshold based on the Matthews correlation coefficient are passed to Stage 2 as structured evidence. This strategy aims to suppress low-confidence predictions and reduce the risk of propagating noisy or spurious findings into the multimodal reasoning process.

To evaluate whether incorporating explicit uncertainty information could further enhance robustness, we introduce a probability-aware setting. Specifically, instead of only providing the predicted recognition findings, we integrate all 36 radiological findings, along with their corresponding probability scores provided by the recognition model, into Stage 2. At the same time, all other components of the framework—including the Stage 1 model, the retrieval-augmented generation (RAG) module, patient demographic information, and the prompting strategy—remain unchanged to ensure that any performance variation can be attributed solely to the probabilities integration.

The quantitative comparison results are summarized in Table 7. Compared with the original framework, incorporating full probability information leads to a consistent decrease across all evaluation metrics. Specifically, accuracy decreases from 0.879 to 0.866, F1-score decreases from 0.782 to 0.763, and AUC drops substantially from 0.808 to 0.678, proving the robustness of the settings in the proposed framework.

### 4.5. Computational Efficiency

Beyond diagnostic accuracy and ablation analysis, we further evaluate the computational efficiency of the proposed framework to assess its feasibility for real-world clinical deployment. Specifically, we report the end-to-end inference time of the two-stage pipeline, including radiological finding recognition and retrieval-augmented MLLM reasoning. The average processing time per sample is measured over the entire test set. The detailed results are summarized in Table 8, where the unit applied is second (s). As can be seen form the results, the inference time is always under 10 s, showing that our framework could give a referrable diagnosis output for clinical doctors in a very short time, which proves the feasibility of the designed method.

## 5. Discussion and Conclusions

### 5.1. Results Analysis and Discussion

In this study, we propose a two-stage paradigm for pediatric radiological diagnosis that aligns closely with real-world clinical workflows. The proposed framework leverages a vision–language model (VLM) for radiological finding recognition and a multimodal large language model (MLLM) for disease diagnosis, explicitly separating perception and reasoning stages. By decoupling radiological finding recognition from disease diagnosis and grounding the diagnostic process in authoritative medical knowledge, the framework achieves robust and interpretable performance on the VinDr-PCXR dataset.

The experimental results demonstrate that integrating structured visual evidence with MLLMs is an effective strategy for addressing key challenges in pediatric radiology, including data scarcity and diagnostic ambiguity. Overall, the proposed framework consistently outperforms existing deep learning-based and LLM-based baselines across most evaluation metrics. In particular, the explicit provision of suspected radiological findings generated by the fine-tuned VLM significantly improves the interpretability and reliability of the diagnostic process compared with traditional black-box deep learning approaches. This structured evidence also helps mitigate hallucination which is commonly observed in MLLMs’ outputs. Furthermore, the retrieval-augmented generation (RAG) module transforms the diagnostic process into a knowledge-grounded reasoning pipeline, enabling the model to align its predictions with established clinical guidelines. The ablation study further confirms the necessity of each module: ablating radiological findings, demographic information, or external clinical knowledge leads to a noticeable degradation in performance, indicating that these components play complementary and indispensable roles in the overall framework.

Nevertheless, we observe that the recognition performance in Stage 1 does not achieve optimal accuracy across all radiological findings. Although the fine-tuned KAD model demonstrates relatively high precision—thereby limiting the risk of introducing misleading evidence—imperfect recognition inevitably propagates some noise to the downstream diagnostic stage. This limitation suggests that further improvements in radiological finding recognition, potentially through more specialized architectures or larger pediatric datasets, could further enhance the overall system performance.

Regarding the choice of MLLMs, the comparative analysis reveals that model selection has a substantial impact on diagnostic outcomes. GPT-4o consistently achieves the best performance, followed by Claude-3.5-sonnet and Gemini-2.5-pro respectively. This performance gap indicates that strong multimodal alignment, robust instruction-following capability, and effective integration of external knowledge are critical factors for reliable medical diagnosis. These findings also suggest that not all MLLMs are equally suitable for clinical applications, underscoring the importance of careful model selection. In the future, we would test our paradigm based on more MLLMs to find out the most suitable model if available.

A more detailed class-wise analysis further reveals both strengths and limitations of the proposed framework. For the “No finding” category, which corresponds to normal patients without respiratory disease, the framework achieves high recall—an especially desirable property in clinical screening scenarios. The framework also demonstrates relatively stable performance across different MLLMs for common conditions such as bronchitis and bronchiolitis. However, for disease categories with similar radiographic manifestations, such as pneumonia and broncho-pneumonia, the recall remains comparatively lower. This limitation can be attributed to both class imbalance in the dataset and the intrinsic visual similarity between these conditions. Addressing class imbalance and improving discrimination between similar diseases will be an important direction for future optimization.

### 5.2. Clinical Application Significance

From a clinical perspective, the proposed framework is not intended to replace radiologists or pediatricians, but rather to function as a trustworthy decision-support system that complements clinical expertise, particularly in resource-limited environments. Increasing evidence suggests that successful deployment of AI in radiology depends not only on predictive accuracy but also on interpretability, transparency, and workflow compatibility [39]. In existing radiological AI studies, interpretability is often presented through post hoc visualization techniques such as Grad-CAM, which provide pixel-level saliency maps to highlight image regions associated with predictions [40,41]. While such spatial explanations enhance transparency, they primarily offer image-level attribution but do not explicitly model the structured diagnostic reasoning process underlying clinical decision-making.

Additionally, explanations that align more closely with clinical reasoning patterns tend to improve user confidence compared with superficial visual overlays alone [42]. In this context, our evidence-guided paradigm explicitly separates radiological finding recognition from disease inference and reasoning stage, enabling clinicians to verify intermediate findings and understand how final diagnostic hypotheses are formed.

Regarding practical deployment, the end-to-end inference latency remains within a clinically acceptable range. The complete two-stage pipeline requires approximately 6–9 s per case depending on the selected MLLM. Although suitable for decision-support scenarios, further optimization would be required for time-critical emergency applications.

When deploying large language model-based systems in clinical environments, ethical and regulatory alignment are essential. The system must ensure patient data privacy in compliance with HIPAA (The Health Insurance Portability and Accountability Act) and regional pediatric data protection guidelines, particularly when interacting with external data retrieval components.

### 5.3. Limitations and Future Directions

Despite the strengths mentioned above, several limitations should be acknowledged. First, although our experiments are conducted on publicly available pediatric chest X-ray datasets, such datasets remain limited in scale, diversity, and disease coverage. In particular, due to severe class imbalance and the scarcity of certain rare pediatric conditions, infrequent categories were consolidated into an “Other diseases” class. While this strategy improves statistical stability and ensures fair comparison across methods, it inevitably reduces diagnostic granularity and may obscure subtle yet clinically meaningful distinctions among rare entities. Future validation on larger, multi-institutional pediatric cohorts with more comprehensive annotations will be essential to enable finer diagnostic stratification and to further assess real-world generalizability across heterogeneous clinical settings.

Second, the external medical knowledge injected via the vector database is manually curated and thus limited in scale and update frequency. Although the incorporated sources are authoritative and clinically reviewed, this static knowledge construction may not fully capture the evolving landscape of pediatric radiology. In the future, we intend to enrich the multimodal knowledge base by incorporating structured clinical narratives, laboratory indicators, and longitudinal imaging records. Combining continual learning strategies and wider external knowledge will allow the system to dynamically adapt to evolving medical standards and new disease categories. Expanding the knowledge base through semi-automated literature mining, guideline integration, and real-world case incorporation could further enhance diagnostic robustness and long-term reproducibility.

In clinical practice, multiple imaging modalities are considered for diagnosis, rather than relying solely on radiographs. The proposed two-stage, evidence-guided reasoning design can be generalized to other pediatric imaging areas such as abdominal ultrasound, brain MRI, and laboratory test data. This extension would test the framework’s universality and support the development of a multimodal pediatric diagnostic assistant across a broader range of disease categories.

Finally, to move beyond retrospective experiments, we aim to implement the proposed system within real hospital workflows and conduct prospective validation studies. These pilot deployments will capture real-time clinician feedback, enabling assessment of interpretability, efficiency, and practical impacts on the pediatric diagnostic process, thereby informing future improvement.

### 5.4. Conclusions

In this work, we propose an evidence-guided, two-stage diagnostic framework for pediatric chest radiography that explicitly aligns with real-world clinical workflows. By first recognizing radiological findings through a domain-adapted vision–language model and subsequently performing knowledge-augmented reasoning with a multimodal large language model (MLLM), the framework enhances interpretability, traceability, and clinical credibility.

The first stage leverages transfer learning from adult radiology pretraining to mitigate pediatric data scarcity, enabling robust recognition of fine-grained radiological findings. The second stage integrates multimodal patient information. Through a retrieval-augmented generation (RAG) mechanism, authoritative pediatric radiology guidelines and other knowledge are dynamically retrieved and incorporated into the diagnostic reasoning process. This design ensures that diagnostic conclusions are explicitly grounded in established medical evidence, reducing the risk of hallucinated outputs and improving decision reliability.

Extensive experiments on the VinDr-PCXR dataset demonstrate that the proposed framework achieves state-of-the-art performance, reaching 90.1% diagnostic accuracy and 82.5% AUC. These results validate the effectiveness of combining structured radiological evidence with knowledge-enhanced multimodal reasoning, particularly in data-constrained pediatric scenarios.

In future research, besides carrying out validation on wider datasets from multi-centers, we plan to extend the framework to additional pediatric imaging modalities and investigate adaptive knowledge updating mechanisms for continuously evolving clinical guidelines. We believe that the proposed evidence-guided, workflow-aligned diagnostic paradigm demonstrates potential for clinical integration.

## Figures and Tables

**Table 1:** Summary of the dataset statistics.

	Training	Testing
Number of samples	7728	1397
Local labels	36	18
Global labels	16	12
Male (%)	57.63	59.14
Female (%)	42.37	40.86

**Table 2:** Comparison of different methods on Stage 1 recognition performance on VinDr-PCXR.

Method	Accuracy	Precision	Recall	F1-Score
ViT-B/16	0.420	0.063	0.595	0.114
GPT-4o	0.839	0.411	0.336	0.232
KAD w/o SFT	0.556	0.120	0.670	0.080
**KAD SFT**	**0.895**	**0.635**	**0.569**	**0.586**

The bolded values represent the highest scores for this indicator.

**Table 3:** Comparison results of the proposed framework and other methods on the VinDr-PCXR dataset on the overall diagnosis.

Method	Accuracy	Precision	Recall	F1-Score	AUC
D-CNN [17]	0.644	0.635	0.649	0.642	N/A
Three-stage [35]	0.770	0.279	0.691	0.376	N/A
Dual-MAE [36]	N/A	0.563	0.715	0.593	0.752
CurriMAE [37]	N/A	0.556	**0.759**	0.622	0.756
ViT-B/16 [38]	0.644	0.635	0.649	0.642	N/A
**Ours-GPT-4o**	**0.901**	**0.709**	0.710	**0.709**	**0.825**
Ours-Gemini-2.5-pro	0.833	0.510	0.513	0.511	0.706
Ours-Claude3-5-sonnet	0.842	0.535	0.534	0.534	0.720

The bolded values represent the highest scores for this indicator.

**Table 4:** Performance comparison of different MLLMs in each category.

Class	Method	Accuracy	Precision	Recall	F1-Score	AUC
Bronchitis	GPT-4o	**0.904**	**0.773**	0.370	0.500	0.677
	Claude3-5-sonnet	0.892	0.578	0.444	**0.503**	0.700
	Gemini-2.5-pro	0.873	0.400	**0.667**	0.500	**0.781**
Broncho-pneumonia	GPT-4o	**0.910**	**0.421**	0.615	**0.500**	**0.774**
	Claude3-5-sonnet	0.723	0.120	0.566	0.198	0.650
	Gemini-2.5-pro	0.757	0.208	**0.733**	0.324	0.746
Bronchiolitis	GPT-4o	**0.907**	**0.452**	**0.655**	**0.535**	**0.792**
	Claude3-5-sonnet	0.892	0.293	0.393	0.336	0.661
	Gemini-2.5-pro	0.804	0.154	0.600	0.245	0.708
Pneumonia	GPT-4o	0.938	0.636	0.280	0.389	0.634
	Claude3-5-sonnet	0.952	**0.842**	0.291	0.432	0.644
	Gemini-2.5-pro	**0.979**	0.833	**0.833**	**0.833**	**0.911**
Other disease	GPT-4o	0.952	**1.000**	0.292	0.452	0.646
	Claude3-5-sonnet	0.942	0.545	0.115	0.191	0.555
	Gemini-2.5-pro	**0.974**	**1.000**	**0.643**	**0.783**	**0.821**
No finding	GPT-4o	**0.797**	0.789	**0.901**	**0.841**	**0.771**
	Claude3-5-sonnet	0.653	0.790	0.627	0.699	0.664
	Gemini-2.5-pro	0.614	**1.000**	0.411	0.583	0.706

The bolded values represent the highest scores for this indicator.

**Table 5:** Results of ablation study under different settings.

Setting	Accuracy	Precision	Recall	F1-Score	AUC
Direct prompting	0.743	0.240	0.236	0.238	0.541
w/o RAG	0.870	0.620	0.608	0.614	0.766
w/o findings	0.878	0.653	0.635	0.634	0.782
w/o Age & Sex	0.873	0.630	0.618	0.624	0.772
**Entire framework**	**0.879**	**0.784**	**0.779**	**0.782**	**0.808**

The bolded values represent the highest scores for this indicator.

**Table 6:** Comparison of different Top-k retrieval configurations in the RAG module.

Top-k	Accuracy	Precision	Recall	F1-Score	AUC
Top-1	0.874	0.778	0.773	0.775	0.768
Top-2	0.875	0.781	0.776	0.778	0.776
**Top-3**	**0.879**	**0.784**	**0.779**	**0.782**	**0.808**
Top-4	0.872	0.775	0.771	0.773	0.663
Top-5	0.871	0.772	0.767	0.769	0.561

The bolded values represent the highest scores for this indicator.

**Table 7:** Ablation results of probability integration.

Probability Setting	Accuracy	Precision	Recall	F1-Score	AUC
Original framework	**0.879**	**0.784**	**0.779**	**0.782**	**0.808**
All findings with probs	0.866	0.765	0.761	0.763	0.678

The bolded values represent the highest scores for this indicator.

**Table 8:** End-to-end inference time analysis of the proposed two-stage framework.

Stage 2 Model	Stage 1 (s)	Stage 2 (s)	Retrieval (s)	LLM (s)	Overall (s)
GPT-4o	0.0987	6.11	2.20	3.92	6.21
Gemini-2.5-Pro	0.0987	9.24	2.65	6.48	9.34
Claude-3.5-Sonnet	0.0987	7.33	2.34	5.90	7.43

## Data Availability

The original data presented in the study are openly available at [29].
